# Exploring Coronavirus Disease 2019 and Brugada Syndrome

**DOI:** 10.19102/icrm.2022.130607

**Published:** 2022-06-15

**Keywords:** Brugada syndrome, COVID-19, electrical storm, fever

## Dr. Brugada discusses

Ali and Nilsson present in this issue of *The Journal of Innovations in Cardiac Rhythm Management* the case of a patient with Brugada syndrome (BrS) with electrical storm triggered by fever.^1^ By itself, nothing new, one might think. The fact that fever worsens the electrocardiogram pattern of BrS and may trigger the ventricular arrhythmias (VAs) has been known for a while.^2^ The sodium (Na^+^) channel mutations behind the temperature dependence of this phenomenon have been characterized.^3^ However, there are a few interesting things in this patient: (1) his age (73 years), (2) the clinical presentation of the in-hospital arrhythmia storm, and (3) the possible underlying mechanisms in the setting of coronavirus disease (COVID-19).

First, the temperature dependence of Na^+^ channel mutations in BrS has been demonstrated in vitro.^3^ Many clinical correlates for this phenomenon have been found, mostly in children. To the best of my knowledge, no case has even been reported in septuagenarians. Thus, this case makes us aware of the potential harmful effects of fever not only in children but also adults with BrS.

In this case, a minimal rise in body temperature triggered the in-hospital, well-documented electrical storm. This phenomenon, known from other diseases such as periodic paralysis,^4^ illustrates that it is the velocity of the rise of temperature and not the temperature level, “per se,” that triggers the arrhythmias. This suggests that some current recommendations about fever in patients with BrS are incorrect: it is usually recommended to aggressively treat fever in BrS when it reaches a certain level (I do not know of any such published recommendations, but many physicians and hospitals recommend doing so in BrS as seen in patients’ discharge letters). The observations in this patient bring a note of caution about the recommendations. Fever, whatever the level, is always a potential problem in BrS.

Most of the emphasis on COVID-19 has been on pulmonary and systemic complications. However, it is well known that cardiac affectation and complications are a major source of morbidity and mortality. Troponin levels at the time of admission are an excellent marker of possible complications and the prognosis of COVID-19.^5^ Increased troponin levels are the result of cardiac inflammation and cellular death. The question is whether a component of myocarditis can also play a role in the arrhythmias documented in this patient. This hypothesis will not seem that strange if one recalls that the right ventricular epicardium is involved in the mechanisms of arrhythmias in BrS.^6^ A perimyocarditis in that area could further slow conduction in and around the BrS substrate, or further accentuate the differences in epicardial and endocardial action potentials, and trigger the VAs alone or together with the fever component.

This case, an apparently simple case of VAs triggered by fever in BrS, turns out to engender many points of discussion and thoughts to ponder. It is clear that much remains to be learned.

My congratulations to the authors for this great contribution.

Pedro Brugada, MD, PhD (pedro@brugada.org)^1^

^1^Cardiovascular Division, UZ Brussel-VUB, Brussels, Belgium

The author reports no conflicts of interest for the published content. No funding information was provided.

References1.AliSHNilssonKRElectrical storm in a patient with Brugada syndrome and coronavirus disease 2019J Innov Card Rhythm Manag20221365019502310.19102/icrm.2022.130601PMC9221185357655842.AdlerATopazGHellerKFever-induced Brugada pattern: how common is it and what does it mean?Heart Rhythm201310913751382[CrossRef]
[PubMed]2387269110.1016/j.hrthm.2013.07.030PMC38327403.KellerDIRougierJSKuceraJPBrugada syndrome and fever: genetic and molecular characterization of patients carrying SCN5A mutationsCardiovasc Res2005673510519[CrossRef]
[PubMed]1589032310.1016/j.cardiores.2005.03.0244.GargRKMalhotraHSVermaRSharmaPSinghMKEtiological spectrum of hypokalemic paralysis: a retrospective analysis of 29 patientsAnn Indian Acad Neurol2013163365370[CrossRef]
[PubMed]2410181810.4103/0972-2327.116934PMC37882825.SalvaticiMBarbieriBCioffiSAssociation between cardiac troponin I and mortality in patients with COVID-19Biomarkers2020258634640[CrossRef]
[PubMed]3300396110.1080/1354750X.2020.1831609PMC77117286.NademaneeKVeerakulGChandanamatthaPPrevention of ventricular fibrillation episodes in Brugada syndrome by catheter ablation over the anterior right ventricular outflow tract epicardiumCirculation20111231212701279[CrossRef]
[PubMed]2140309810.1161/CIRCULATIONAHA.110.972612

## Drs. Wu and Wilde consider

Patients with Brugada syndrome (BrS) might be vulnerable to febrile status and suffer from associated lethal arrhythmia events (LAEs). In the era of the COVID-19 pandemic, pyrexia is such a common symptom as patients become infected by severe acute respiratory syndrome coronavirus 2 (SARS-CoV-2).^1^ Increased body temperature decreases SCN5a channel conductance, thereby increasing the severity of the phenotype, with potential proarrhythmic consequences. Indeed, BrS patients are at risk during fever,^2^ and this may be particularly relevant for carriers of a pathogenic loss-of-function sodium (Na^+^) channel variant (as demonstrated in children).^3^ In other words, patients with BrS are put at risk after being infected by this virus.

LAEs include ventricular fibrillation or fast ventricular tachycardias. When VAs happen to a patient >3 times within 24 h, it is defined as an electrical storm, which has been well described in the case report by Ali and Nilsson.^4^ In order to prevent these arrhythmias, first of all, aggressive management of anti-pyretics is of importance. This case report not only reminds us of the crucial role of anti-pyretics management, but the authors also emphasize the importance of associated pharmacological therapies in this unique situation. However, before particularizing these managements, it is important to highlight several aspects as to this patient’s presentation.

First of all, the electrocardiogram (ECG) (**Figure 1** in the report by Ali and Nilsson) represents, at best, an atypical Brugada ECG pattern because it lacks a true coved-type ST-segment elevation (type 1). The definite type 1 ECG pattern is supposed to have an elevated J point or ST segment that is >2 mm and should be followed by an inverted T-wave.^5,6^ A reason for the lack of true ST-segment elevation in this particular case may be the right bundle branch block (wide S in the lateral leads), which seems to be apparent and which masks a true Brugada pattern.^7^ Further, the origin of the arrhythmia, as demonstrated in **Figure 2** of their paper (leads I, II, and III are presumably depicted), could be the right ventricular outflow tract area, as one would expect in a BrS patient,^8^ but there is no clear inferior axis. Finally, the presence of the P–R interval prolongation (>200 ms) and the fragmented QRS complex (V_2_) in this ECG suggests that this patient harbors a loss-of-function Na^+^ channel variant, but that is not reported. The corrected QT interval (QTc) seems somewhat prolonged, but the widened QRS complex contributes to this. The use of quinidine may also, in part, contribute to the relatively long QTc. The documented arrhythmia **(Figure 2)**, however, is not related to the eventual QT prolongation as the coupling interval of the initiating extrasystole is short and there is no preceding pause as one would expect in the case of quinidine proarrhythmogeneity. The other 2 drugs that were prescribed before the arrhythmia, ie, doxycycline and dexamethasone, are not expected to impact the electrophysiological properties of the heart. Key in the management of an arrhythmic storm in this condition is to remove an eventual external trigger, ie, the elevated body temperature. Hence, aggressive anti-pyretic treatment is critical. Furthermore, intravenous isoproterenol is the medication of choice. Indeed, intravenous isoproterenol has been shown to be effective in terminating an arrhythmic storm in, respectively, 6 of 7 and all of 5 patients where this intervention was described.^9,10^ An alternative or additional approach is cilostazol.

Cilostazol is a phosphodiesterase type III inhibitor. The primary mechanism of this medicine is to inhibit the function of platelets, but this drug can also increase impacts on cardiac ion channels, in particular inhibition of the transient outward current channel (I_to_) and potentially augmentation of the L-type calcium channel (I_Ca_). Both effects are potentially beneficial in BrS because they will increase the safety of conduction by normalizing the action potential morphology.^11,12^ We see no particular reason, as suggested by the authors, that this is limited to specific subgroups of BrS patients.

Taken together, when patients with BrS were infected by SARS-CoV-2, aggressive anti-pyretics management was of importance.^1^ In addition, isoproterenol, quinidine, and/or cilostazol should be used when there is an arrhythmic storm. Together with the removal of the eventual trigger hyperthermia, these drugs will suppress the potential lethal ventricular arrhythmias. Ultimately, as also mentioned by the authors, external cooling might be needed.

Cheng-I Wu, MD^1,2^ and Arthur A.M. Wilde, MD, PhD (a.a.wilde@amc.uva.nl)^2,3^

^1^Heart Rhythm Center, Department of Cardiology, Taipei Veterans General Hospital, Taipei, Taiwan

^2^Department of Clinical and Experimental Cardiology, Amsterdam University Medical Center, Academic Medical Centre, University of Amsterdam, Amsterdam, The Netherlands

^3^European Reference Network for Rare and Low Prevalence Complex Diseases of the Heart, Amsterdam, The Netherlands

The authors report no conflicts of interest for the published content. No funding information was provided.

References1.WuCIPostemaPGArbeloESARS-CoV-2, COVID-19, and inherited arrhythmia syndromesHeart Rhythm202017914561462[CrossRef]
[PubMed]3224405910.1016/j.hrthm.2020.03.024PMC71561572.MizusawaYMoritaHAdlerAPrognostic significance of fever-induced Brugada syndromeHeart Rhythm201613715151520[CrossRef]
[PubMed]2703363710.1016/j.hrthm.2016.03.0443.PeltenburgPJBlomNAVinkASIn children and adolescents from Brugada syndrome-families, only *SCN5A* mutation carriers develop a type-1 ECG pattern induced by feverCirculation202014218991[CrossRef]
[PubMed]3262855210.1161/CIRCULATIONAHA.120.0457204.AliSHNilssonKRElectrical storm in a patient with Brugada syndrome and coronavirus disease 2019J Innov Card Rhythm Manag20221365019502310.19102/icrm.2022.130601PMC9221185357655845.MizusawaYWildeAAArrhythmogenic disorders of genetic origin: Brugada syndromeCirc Arrhythm Electrophysiol2012536066162271524010.1161/CIRCEP.111.9645776.AntzelevitchCYanGXAckermanMJJ-wave syndromes expert consensus conference report: emerging concepts and gaps in knowledgeJ Arrhythm2016325315339[CrossRef]
[PubMed]2776115510.1016/j.joa.2016.07.002PMC50632707.ChialePAGarroHAFernándezPAElizariMVHigh-degree right bundle branch block obscuring the diagnosis of Brugada electrocardiographic patternHeart Rhythm201296974976[CrossRef]
[PubMed]2230610010.1016/j.hrthm.2012.01.0288.NademaneeKRadiofrequency ablation in Brugada syndromeHeart Rhythm2021181018051806[CrossRef]
[PubMed]3460061110.1016/j.hrthm.2021.08.0059.WatanabeAFukushima KusanoKMoritaHLow-dose isoproterenol for repetitive ventricular arrhythmia in patients with Brugada syndromeEur Heart J2006271315791583[CrossRef]
[PubMed]1676020810.1093/eurheartj/ehl06010.OhgoTOkamuraHNodaTAcute and chronic management in patients with Brugada syndrome associated with electrical storm of ventricular fibrillationHeart Rhythm200746695700[CrossRef]
[PubMed]1755618610.1016/j.hrthm.2007.02.01411.WildeAAPostemaPGDi DiegoJMThe pathophysiological mechanism underlying Brugada syndrome: depolarization versus repolarizationJ Mol Cell Cardiol2010494543553[CrossRef]
[PubMed]2065947510.1016/j.yjmcc.2010.07.012PMC293280612.BehrERBen-HaimYAckermanMJKrahnADWildeAAMBrugada syndrome and reduced right ventricular outflow tract conduction reserve: a final common pathway?Eur Heart J2021421110731081[CrossRef]
[PubMed]3342105110.1093/eurheartj/ehaa1051

## Drs. Baranchuk, Alexander, and Miranda-Arboleda examine

BrS and its interaction with fever is a well-established physiopathological phenomenon.^1^ Since the cornerstone work of Antzelevitch and Brugada,^2^ fever has emerged as a trigger to unmask a possible concealed Brugada ECG pattern, and, more importantly, potentially increases the risk of triggering VAs due to alterations at the ionic level.^2^ Sodium channels are thermo-sensitive, and high temperatures can distort normal functioning, predisposing the patient to action potential alterations that are the physiopathological substrate for the development of VAs.^1,2^

In this issue of *The Journal of Innovations in Cardiac Rhythm Management*, Ali and Nilsson^3^ describe an interesting case of a 73-year-old man with a proven history of BrS who presented with COVID-19 pneumonia and subsequently developed VA constituting an arrhythmia storm. He was successfully treated by his implantable cardioverter-defibrillator (ICD), and clinical management was oriented to reduce the arrhythmia recurrence with quinidine and energic anti-pyretic treatment. Evolution was favorable.^3^

BrS and fever in the context of viral infections has been previously reported in the context of H1N1 infections^4^ and may follow the same behavior in the case of exposure to SARS-CoV-2. The unmasking of BrS by fever and the triggering of VAs by fever in patients with an established diagnosis of BrS are 2 well-documented phenomena.^1–4^

The case by Ali and Nilsson^3^ should be highlighted given the high volume of patients presenting with COVID-19 worldwide, which may increase the number of patients seen with BrS and fever. A special emphasis in normalizing body temperature should be placed.^3^

Some minor discrepancies with terminology used through the paper are worth mentioning: BrS ECG pattern does not present with right bundle branch block (RBBB).^5^ The initial description mentions RBBB; however, this was corrected over time using vectorcardiography to support the lack of distal conduction delay in the right bundle.^5^ Cases of simultaneous presentation of BrS and transient RBBB were published by different groups, and electrophysiological maneuvers to unmask BrS ECG pattern in cases of RBBB were also described.^6^ We should update definitions, such as the ones used in the third consensus on BrS by Bayés de Luna et al.,^7^ which were adopted in the present report. In concordance with this document, the BrS ECG patterns were reduced to 2 types only (“coved” and “saddleback”), as the initially described type 3 does not lead to the diagnosis of BrS.^7^

In order to confirm that the only identifiable trigger was fever, in such a case, the laboratory work done to rule out metabolic and electrolyte disturbances, also well-known factors that could potentially contribute to VA storm,^3^ should be reported.

We would like to conclude with some speculations about COVID-19 and myocardial injury in the context of BrS.^8^ Given that fever can modulate ion channels, it is thought that VA induction is attributed to fever, rather than to an inflammatory myocardial reaction in the presence of SARS-CoV-2.^1,2,8^ As in any other viral infections, myocarditis, myocardial injury, and excessive inflammatory response can also play a role in the genesis of VAs.^8^ In this sense, follow-up with cardiac magnetic resonance imaging (considering appropriate programming of the ICD) could be recommended.^8^

Ali and Nelsson^3^ should be commended for bringing us such a wonderful case with great illustrations.

Adrian Baranchuk, MD (barancha@kgh.kari.net),^1^ Bryce Alexander, MD,^1^ and Andres Felipe Miranda-Arboleda, MD^1^

^1^Division of Cardiology, Kingston Health Science Center, Queen’s University, Kingston, Ontario, Canada

The authors report no conflicts of interest for the published content. No funding information was provided.
